# A New Definition of Aging?

**DOI:** 10.3389/fgene.2012.00148

**Published:** 2012-08-23

**Authors:** Thomas Flatt

**Affiliations:** ^1^Department of Biomedical Sciences, Institute of Population GeneticsVetmeduni Vienna, Vienna, Austria

I do not fundamentally differ with Michael Rose's definition of aging and his thesis. For him aging is defined as a decline or loss (a “de-tuning”) of adaptation with increasing age, caused by a time-progressive decline of Hamilton's forces of natural selection. To my mind, this definition is consistent with most previous definitions of aging used by the majority of evolutionary biologists.

Most evolutionary biologists define aging as an age-dependent or age-progressive decline in intrinsic physiological function, leading to an increase in age-specific mortality rate (i.e., a decrease in survival rate) and a decrease in age-specific reproductive rate (e.g., Medawar, 1955; Williams, 1957; Rose, 1991; Partridge and Barton, 1996; Tatar, 2001; Promislow and Bronikowski, 2006; Flatt and Schmidt, 2009; Bronikowski and Flatt, 2010; Fabian and Flatt, 2011). Rose (1991), for example, in his seminal book on the evolution of aging defines aging as “*a persistent decline in the age-specific fitness components of an organism due to internal physiological degeneration*.” At level of the individual, the intrinsic physiological state at a specific age determines, among other things, whether an individual is dead or alive and how much it reproduces. At the level of the cohort, the underlying physiological states of the individuals translate into the age-specific rates of mortality and reproduction. We therefore diagnose demographic aging to occur if we observe an increase in age-specific mortality and a decrease in age-specific reproductive rate in the cohort (e.g., Bronikowski and Flatt, 2010).

In particular, the notion that aging is a de-tuning of adaptation is completely consistent with the standard definition of aging. Adaptation by natural selection is based on heritable variation in fitness among individuals, and survival and reproduction are the most important determinants or components of fitness (e.g., Stearns, 1992). Thus, in agreement with Rose's definition, the age-dependent decline of age-specific survival and reproductive rates represents an age-progressive loss or de-tuning of fitness or the *state of adaptation*. Moreover, it is well-accepted among evolutionary biologists that this decline is due to the declining forces of natural selection with increasing age. So where exactly do Rose's definition and main thesis differ from the standard definition of aging?

The crux of Rose's thesis is not so much about the fact that adaptation and the forces of selection *decline* but about the fact that the forces *plateau* (Rose et al., 2007). Although this is well-known from both theory and data, the important point is that, under protected conditions (low extrinsic mortality), age-specific mortality and fecundity *also* exhibit plateaus late in life. This means that the age-specific rates of survival and reproduction do not necessarily decrease to zero toward the end of life, as had previously (mostly implicitly yet incorrectly) been assumed. In fact, the existence of such plateaus has often been missed because observing them requires very large cohort sizes. The work by Rose and others now posits that these late life mortality and fecundity plateaus are a direct, causal consequence of the plateaus in the forces of selection.

The real issue at stake is therefore that the aging process is not sufficiently well defined if we describe it as a *persistent* age-progressive decline of physiological function leading to a *steady* age-progressive decline in survival and reproduction. Instead, demographic aging involves an age-progressive decline in survival and reproduction which is often (perhaps always) being followed by plateaus in the rates of mortality and reproduction. In other words, the age-dependent decline that is characteristic of the aging process is therefore not (at least not necessarily) persistent and steady but levels off. Note Rose's (1991) use of the word “*persistent*” in his classical definition of aging quoted above; clearly, in view of the by now well-documented demographic cessation of aging at advanced ages and under protected conditions, the usage of the term “persistent” is problematic. However, this being said, it should be pointed out that most evolutionary biologists do not include terms such as “persistent” or “steady” in their definitions of aging: most of them simply characterize demographic aging as an age-progressive increase in mortality rate and a decrease in reproductive rate. From my point of view, such a definition of aging is certainly not wrong, but one might say that it is not sufficiently precise. Obviously, in any finite cohort of individuals, the age-specific rates of survival and reproduction will eventually reach zero at some point of time: everyone has died and stopped reproducing. Thus, survival and reproduction *do decline* to zero levels *in a time-progressive manner*. However, as the existence of plateaus demonstrates, it would be wrong to assume that these rates decline *steadily* toward zero. If it turns out that such plateaus are general under benign conditions (the evidence so far suggests that they might be), then their existence must be integrated into the standard definition of the aging process.

Similar considerations apply when we talk about the age-progressive decline of physiological function. We might still say that demographic aging at the cohort level reflects the age-dependent decline or deterioration of physiological function of the individuals in the cohort; however, we should not take this to mean that physiological function deteriorates *steadily* toward zero functionality. In a finite population with individuals of finite lifespan, physiological functionality will be zero at some point; however, before this point is reached, it might plateau. Although we still know next to nothing about the mechanistic details of physiology during late life, the plateauing of physiological decline might be a direct consequence of the plateaus of Hamilton's forces of natural selection. Thus, if Rose's model is correct, plateaus in the forces of selection cause plateaus in individual physiological state which in turn cause plateaus in mortality and reproduction.

The notion that Hamiltons’ forces of natural selection plateau thus has major implications for our understanding of the aging process and, in particular, of late life: at advanced ages natural selection is unable to distinguish among individuals, be it at the level of physiological state or at the level of fitness components, so that demographic aging, under benign conditions, can come to a halt. While it remains to be seen how general the existence of such plateaus is across species, and although more theoretical and empirical efforts are required to fully understand the nature of the demographic cessation of aging, the phenomenon itself is now empirically well established.
